# Synthesis and Characterization of Covalently Crosslinked pH-Responsive Hyaluronic Acid Nanogels: Effect of Synthesis Parameters

**DOI:** 10.3390/polym11040742

**Published:** 2019-04-24

**Authors:** Sheila Maiz-Fernández, Leyre Pérez-Álvarez, Leire Ruiz-Rubio, Raúl Pérez González, Virginia Sáez-Martínez, Jesica Ruiz Pérez, José Luis Vilas-Vilela

**Affiliations:** 1 Macromolecular Chemistry Group (LABQUIMAC), Department of Physical Chemistry, Faculty of Science and Technology, University of the Basque Country, UPV/EHU, Barrio Sarriena, s/n 48940 Leioa, Spain; sheilamaiz26@gmail.com (S.M.-F.); leire.ruiz@ehu.eus (L.R.-R.); joseluis.vilas@ehu.es (J.L.V.-V.); 2 BCMaterials, Basque Center for Materials, Applications and Nanostructures, UPV/EHU Science Park, 48940 Leioa, Spain; 3 i+Med S. Coop. Parque Tecnológico de Alava. Albert Einstein 15, nave 15. 01510 Miñano, Spain; rperez@imasmed.com (R.P.G.); vsaez@imasmed.com (V.S.-M.); jruiz@imasmed.com (J.R.P.)

**Keywords:** hyaluronic acid, nanogels, divinyl sulfone, 1,4-butanediol diglycidyl ether, poly(ethylene glycol) bis(amine)

## Abstract

Stable hyaluronic acid nanogels were obtained following the water-in-oil microemulsion method by covalent crosslinking with three biocompatible crosslinking agents: Divinyl sulfone, 1,4-butanediol diglycidyl ether (BDDE), and poly(ethylene glycol) bis(amine). All nanoparticles showed a pH-sensitive swelling behavior, according to the pKa value of hyaluronic acid, as a consequence of the ionization of the carboxylic moieties, as it was corroborated by zeta potential measurements. QELS studies were carried out to study the influence of the chemical structure of the crosslinking agents on the particle size of the obtained nanogels. In addition, the effect of the molecular weight of the biopolymer and the degree of crosslinking on the nanogels dimensions was also evaluated for BDDE crosslinked nanoparticles, which showed the highest pH-responsive response.

## 1. Introduction

The majority of drugs display very poor aqueous solubility, resulting in poor bioavailability and pharmacokinetics in vivo [1,2,3]. For this reason, the load of water-insoluble compounds in delivery carriers is always an important issue. It is useful to obtain well-dispersed compounds to overcome their poor solubility in an aqueous solution and this, in turn, may provide a novel path to improve the uptake efficiency in vivo. When considering the design of a nanocarrier, several important factors need to be addressed. An ideal delivery system should be composed of biocompatible and biodegradable materials, which, reproducibly assembled into the desired size range, would be able to encapsulate a wide range of active compounds, maintain stability in biological media, and release the therapeutic at the site of disease.

Nanogels are three-dimensional hydrogel nanoparticles based on crosslinked hydrophilic polymeric networks that have become very promising materials for drug delivery due to their relatively high encapsulation capacity. These swellable polymer networks have a high capacity to hold water (i.e. body fluids), making them generally biocompatible, and do not actually dissolve into the aqueous medium. As soft materials, they are capable of holding molecular therapeutics and active biomolecules, which allows them to find applications for several therapies. Their swelling behaviour and advantageous characteristics such as size, charge, colloidal stability, minimal toxicity, and degradability can be easily controlled by a selection of constituent polymers and crosslinker components.

These nanocarriers protect their cargo from degradation and early elimination, and can also participate actively in the delivery process due to their characteristic swelling properties, to help achieve a controlled, triggered response at the target site. They can be composed of a variety of natural or synthetic polymers, or a combination thereof, with different molecular weights, hydrophilicities, and degradability [4,5]. They determine the chemical structure of the polymer matrix, crosslinking degree, charge density, and swelling/deswelling that usually undergoes in response to external stimuli. This multifunctionality, stability, tunable interior network, and ability to respond to environmental changes enables nanogels to incorporate a broad diversity of actives of very different natures within their structure, and ensures a spatial and stimuli-controlled release of the bioactive compounds as an advantage to other types of nanoparticles. This makes the nanogel an adaptable nanocarrier delivery vehicle, when compared to other delivery systems.

Nanogels are promising materials for biomedical applications. For example, they are able to pass through physiological barriers more easily than stiffer materials, consequently leading to longer circulation half-life. Additionally, the large surface area offers a proper space for functionalization and bioconjugation, while the interior network is suitable for the entrapment (encapsulation) of bioactive substances [6].

There exist various synthetic strategies for the preparation of nanogels using preformed polymers and, via direct polymerization of monomers, using heterogeneous free radical polymerization and heterogeneous controlled/living radical polymerization. The most used strategies are the precipitation polymerization method and the emulsion polymerization method. Both can have disadvantages: In precipitation methods the difference in reactivity and hydrophilicity of monomers may result in a broad size distribution, which might restrict functionalities of resultant particles. In emulsion methods the nanogels may suffer an unstable performance. Due to the lack of standardized synthetic methods to get uniform nanogels with controlled properties, the understanding of the synthesis process’ variables effect on the behavior is still limited, which should be comprehensively considered at design stage [7,8,9,10,11,12,13,14].

Naturally-derived nanogels can be prepared from polysaccharide polymers, such as hyaluronic acid (HA), to be used in drug delivery systems [15,16,17]. HA is a biocompatible, negatively charged linear polysaccharide composed of *N*-acetyl glucosamine and *D*-glucuronic acid as a repeating unit. It is a nontoxic, biologically active, biocompatible, and biodegradable material with unique rheological properties [18]. Furthermore, it contains free carboxylic acid and hydroxyl groups that can be easily modified under mild conditions [19]. HA, one of the main body tissues with lubricant and/or filler functions, is often used alone or crosslinked with other materials to develop different types of biomaterials [20,21]. The natural presence of HA in the human body makes it a promising material with biomedical application. However, HA applicability is limited due to the high viscosity of their solutions and the rapid elimination by the degradation action of hyaluronidase enzymes, among other things.

Nanotechnology proposes new opportunities to overcome these limitations. For instance, HA nanogels have shown higher resistance to enzymatic degradation than uncrosslinked HA [22]. Different methods have been proposed to obtain HA nanoparticles, mainly based on self-assembling or chemical crosslinking [23]. The simplest self-assembling example of HA that leads to the formation of nanoparticles is based on electrostatic interactions with cationic polymers, such as chitosan or proteins. Nevertheless, the instability of these particles against the pH and ionic strength has restricted their applicability. Thus, covalent crosslinking under volume restricted conditions seems to be a more appropriate way to prepare stable HA nanogels.

Although different methods have been employed in HA chemically crosslinked nanogels, such as precipitation or suspension, the most widely used method is the reverse microemulsion crosslinking process in which water-in-oil micelles act as nanoreactors for HA crosslinking reactions, promoting the control of the nanoparticle size. An extensively reported formulation for HA microemulsions is formed by water as an aqueous phase for HA solution, isooctane as an organic phase, and sodium bis (ethylhexyl) sulfosuccinate (AOT) as surfactant, however, Tween 80 and Span 20 [24,25] are also used. Additionally, 1-heptanol is traditionally added as cosurfactant. Regarding crosslinking agents, the use of divinyl sulfone [26,27] (DVS) and 1,4-butanediol diglycidyl ether (BDDE) [28,29] is noteworthy, and, despite having displayed toxicity in their unreacted form for high concentrations [30,31], HA has crosslinked materials containing low concentrations of DVS or BDDE which maintain the biological characteristics of uncrosslinked HA and are approved by the Food and Drug Administration (FDA)) [26,32]. For this reason, new nontoxic molecules are attracting interest as crosslinkers for HA, such as polyethylene glycol (PEG) derivatives [33]. Nonetheless, the toxicity of the crosslinking agent is a minor issue in the formulation of nanogels because they are prepared as highly diluted dispersions. On the other hand, the crosslinking agent plays a crucial role on the synthesis of nanogels because it can influence the particle size and properties of the prepared nanoparticles. Thus, the crosslinker election can be exploited to tailor the final properties of prepared networks.

This paper describes the synthesis and characterization of HA nanogels prepared by crosslinking reactions in the reverse microemulsion medium with different crosslinking agents: Divinyl sulfone (DVS), 1,4-butanediol diglycidyl ether (BDDE), and poly(ethylene glycol) bis(amine) (PEGBNH_2_). The aim of this work is to gain deeper insight into the effect of the crosslinking agent in the particle size, swelling capacity, and pH-responsive behaviour of covalently crosslinked HA nanogels. In addition, the role of the molecular weight and the crosslinking ratio on cited parameters was also analyzed. This study provides helpful conclusions about the control of the particle size and the swelling behaviour of HA nanogels to be used as promising biomaterials for future drug delivery applications.

## 2. Experimental Part

### 2.1. Materials and Chemicals

Hyaluronic acid (HA, sodium salt, 1.2–2.2 MDa, and 500–750 kDa) was purchased from Contripo (nutritional grade). The average molecular weights were measured by gel permeation-chromatography equipped with refractive index (RID) and light scattering (LS15 and LS90) detectors (HPLC Agilent Technologies, Agilent Technologies, Santa Clara, California, USA). Employing a PolySep-GFC-P Linear 300 × 7.8mm Phenomenex column and sodium azide 0.05 M as eluent, with 1 mL/min flow and 20 μL injection volume, followed a detector calibration against a polyethylene oxide narrow standard (1.5 MDa). The obtained values were 2.1 MDa (PDI = 1.009) and 751kDa (PDI = 1.024), respectively. Dioctyl sulfosuccinate sodium salt (Aerosol OT, AOT, 98%), 1-heptanol (1-HP), 2,2,4-trimethylpentane (isooctane), divinyl sulfone (DVS), 1,4-butanediol diglycidyl ether (BDDE), poly(ethylene glycol) bis(amine) (PEGBNH_2_) (6000 g/mol), 1-(3-dimethylaminopropyl)-3ethylcarbodiimide (EDC), *N*-hydroxisuccinimide (NHS), and deuterium oxide (D_2_O) were acquired from Sigma-Aldrich (Saint Louis, Missouri, USA). Ethanol absolute (EtOH) and sodium hydroxide pellets (NaOH) were obtained from Panreac (Damstadt, Germany).

### 2.2. Synthesis of HA Nanogels

HA nanogels were synthesized using water-in-oil microemulsion (W/O) based on the AOT/isooctane/H_2_O system. The aqueous phase was prepared by dissolving HA in 0.2 M NaOH solution at a concentration of 4 mg/mL, while the organic phase, AOT/1-HP/Isooctane (25%/62.5%/12.5%), was separately prepared. The microemulsion was then formed by adding the aqueous phase drop by drop into the organic phase while vigorously stirring. Once a clear system was obtained, the crosslinking agent (DVS or BDDE) was subsequently added, with vigorous stirring, in 1:1 (HA:crosslinker) molar ratio. The mixture was stirred (450–550 rpm) for 1 h at room temperature. Nanoparticles were extracted and purified by successive cycles (×6) of precipitation in ethanol and ultracentrifugation (Eppendorf 5804R, 4 °C, 6000 rpm, 40 min, Sigma-Aldrich, Saint. Louis, MO, USA). Finally, the obtained nanogels were vacuum dried at 60 °C until at a constant weight.

The crosslinking reaction between HA and PEGBNH_2_ required the activation of the carboxylic groups of HA that was carried out by a reaction with the carbodiimide EDC in the presence of NHS. For this, HA and NHS were dissolved in distilled water and the pH was adjusted at 5.4 by an addition of the 2 M NaOH solution. After 15 min of constant stirring, the EDC solid was added slowly until the molar ratio EDC/NHS/COOH was 10:4:1 maintaining the pH at 5.4. The mixture was stirred at room temperature for 4h. After this, the water-in-oil microemulsion method was followed with the HA-PEGBNH_2_ crosslinked nanoparticles.

### 2.3. Determination of the Pseudoternary Phase Diagram

A pseudoternary phase diagram was constructed using the water-in-oil method to find the concentration range of the main components, in which they form microemulsions. The system is based on an aqueous phase (H_2_O) and an organic phase, in which AOT and isooctane can differ. Isooctane and AOT were mixed at a different weight radio and then hyaluronic acid solution was added, dropwise, and with constant agitation. The samples were classified as microemulsions when they appeared as fully transparent liquids (transmittance value up to 60% at λ = 605 nm). It could be noted that 1-heptanol was used as a cosurfactant to interact between the surfactant and water interphase to improve the microemulsion process.

### 2.4. Characterization Methods

#### 2.4.1. Transmission Electron Microscopy (TEM)

The morphological characterization and particle size distribution of prepared nanogels dispersions were studied by using the JEOL JEM 1400 Plus transmission electron microscope (JEOL, Tokyo, Japan). Samples were vacuum dried at 45 °C for 30 h. Dried nanoparticles were dispersed in distilled water (1 mg/mL) and a drop was deposited on a carbon-coated TEM grid that was dried and glow-discharged in a high vacuum chamber. A total of 50 particles were employed for statistical analyses of the particle size in each TEM photograph that lead to obtain particle size distributions. Three samples were analyzed in all the cases, and the average particle sizes (d_p_) and standard deviations could be calculated.

#### 2.4.2. Nuclear Magnetic Resonance (^1^H-NMR)

The compositions of the hyaluronic acid crosslinked nanoparticles were analyzed by NMR spectroscopy. ^1^H-NMR spectra were taken in D_2_O on a Bruker Avance 500MHz spectrometer at 25 °C (Bruker, Billerica, Massachusetts, USA). The samples were prepared using deuterium oxide to disperse the nanogels in a 1 mg/mL concentration.

#### 2.4.3. Dynamic Light Scattering (DLS)

Dynamic light scattering (DLS) allows the measurement of the hydrodynamic size and distribution of nanoparticles. To determine the size distribution of synthesized nanogels, Malvern Zetasizer ZS equipment (Malvern Panalytical, Malvern, UK) was used. The dried powder samples (0.04 mg/mL) were dispersed in distilled water, which swells the crosslinked nanogels resulting in homogeneous dispersions. External pH value was varied by the addition of 0.1 M and 0.01 M NaOH solutions and 1% (v/v) HAc solutions. All measurements were made at room temperature and shown values are the average of 9–12 replicates.

#### 2.4.4. Zeta Potential

Zeta potential measurements were used to analyze the surface charge of the nanogels at different ionization stages and were carried out in a Malvern Zetasizer ZS equipment (Malvern Panalytical, Malvern, UK). Hyaluronic acid nanoparticles were dispersed in distilled water (0.04 mg/mL). Solutions of HAc (1% (v/v) and NaOH (1 M, 0.1 M, 0.01 M) were employed to vary the pH of the medium. The size distributions were obtained by NNLS analysis, and the average size of the obtained distributions are displayed in Figures 5, 7, and 8 where error bars represent the standard deviations of the average size of five different samples.

## 3. Results

### 3.1. Determination of the Microemulsion Phase Diagram

**Figure 1:**
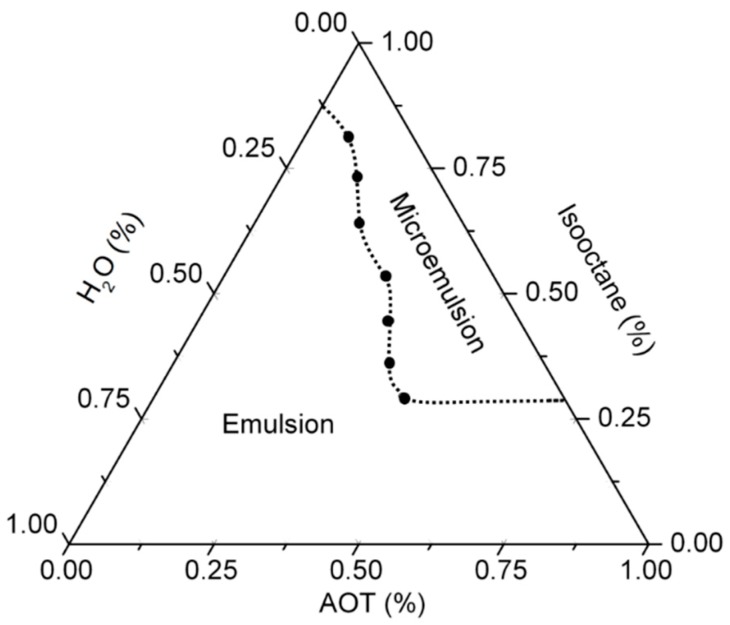
Pseudothernary phase diagram obtained for the system based on H_2_O/ trimethylpentane (isooctane)/ dioctyl sulfosuccinate sodium salt (Aerosol OT, AOT). The white point corresponds with the employed formulation for hyaluronic acid (HA) nanogel preparation.

Figure 1 shows the pseudeternary phase diagram corresponding to the system based on H_2_O (HA)/Isooctane/AOT. The diagram is divided in two areas that separate the microemulsion region (blue) from the emulsion region (red). The area outside the frame of the microemulsion corresponds to the emulsion that indicates a turbid region with the multiphase system. Microemulsions were differentiated from emulsions because they were completely transparent (transmittance up to 60%, at 605 nm) as a consequence of the colloidal size of the micelles, while emulsions were shown to be milky [34,35].

### 3.2. Effect of the Crosslinking Agent

Three crosslinking agents were employed in HA nanogel preparation: DVS, BDDE, and PEGBNH_2_. DVS is a hydrophobic molecule that shows the lowest water-affinity in comparison with the rest of the employed crosslinking agents. Regarding the most hydrophilic molecules, BDDE and PEGBNH_2_, it is worth highlighting the similar chemical structure of both molecules and the significantly longer and flexible chemical structure of PEGBH_2_.

**Figure 2:**
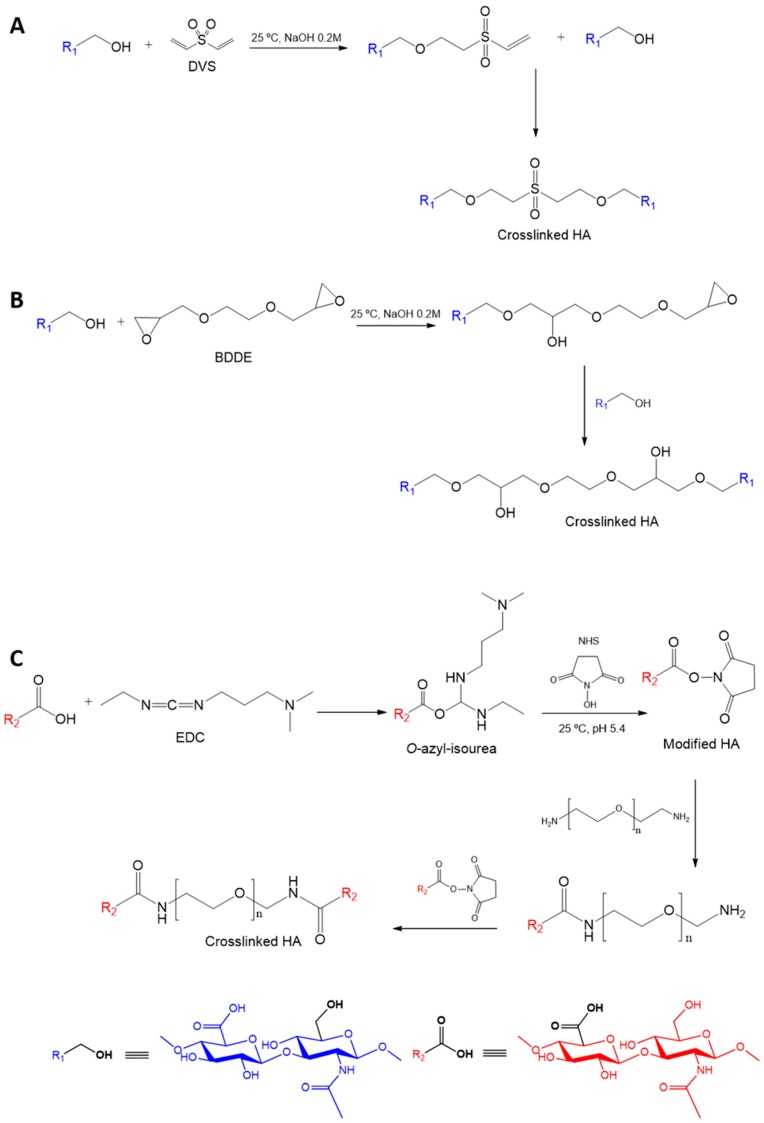
Schemes of crosslinking reactions of HA with (**A**) divinyl sulfone (DVS) (**B**) butanediol diglycidyl ether (BDDE), and (**C**) poly(ethylene glycol) bis(amine) (PEGBNH_2)_ followed in the preparation of covalently crosslinked HA nanogels.

HA presents several functional groups that are suitable for chemical transformation, such as carboxylate, acetamide groups, chain end reducer groups, and hydroxyl groups. Proposed crosslinking agents link to HA by different reaction sites involving different crosslinking pathways (Figure 2). DVS and BDDE, which are commercial covalent crosslinking agents for HA [36,37], present a similar reaction mechanism by reacting with the hydroxyl groups of the polysaccharide. This reaction takes place in basic environments (pH > 11) in which the main hydroxyl groups are ionized (pKa~ 9–10) [38,39], resulting in the formation of ether bonds after the attack of alkoxide groups onto the DVS double bond or BDDE epoxide groups [29], leading to sulphonyl-bis-ethyl or 1,4-butanediol di-(propan-2,3-diolyl) ether bonds, respectively, by Oxa–Michael addiction reactions.

However, the first system reported in the literature about the synthesis of chemically crosslinked HA nanogels followed the strategy of amide coupling by –COOH groups of HA after being initiated by a water soluble carbodiimide [40]. The reaction took place in an emulsified medium by reaction with the –NH_2_ moieties of adipic acid dihydrazide leading to HA microspheres. Since PEG is one of the most popular polymers in the current chemical modification of biomaterials, due to its unique physicochemical and biological properties, such as its high hydrophilicity and biocompatibility, there is a great interest in the use of PEG derivatives as crosslinking agents. In this sense, PEG diamine has emerged in recent years as a promising crosslinker with amide bonds formation for polymers that present carboxylate groups. However, this reaction presents a disadvantage to the previous activation step of HA carboxyl groups that typically take place via a reaction with 1-(3-dimethylaminopropyl) -3-ethylcarbodiimide (EDC) [41], which is highly water-soluble. Commonly, the yield of carboxylate activation reaction is increased using N-hydroxysuccinimide (NHS), which ultimately minimizes side reactions [29,42].

**Figure 3:**
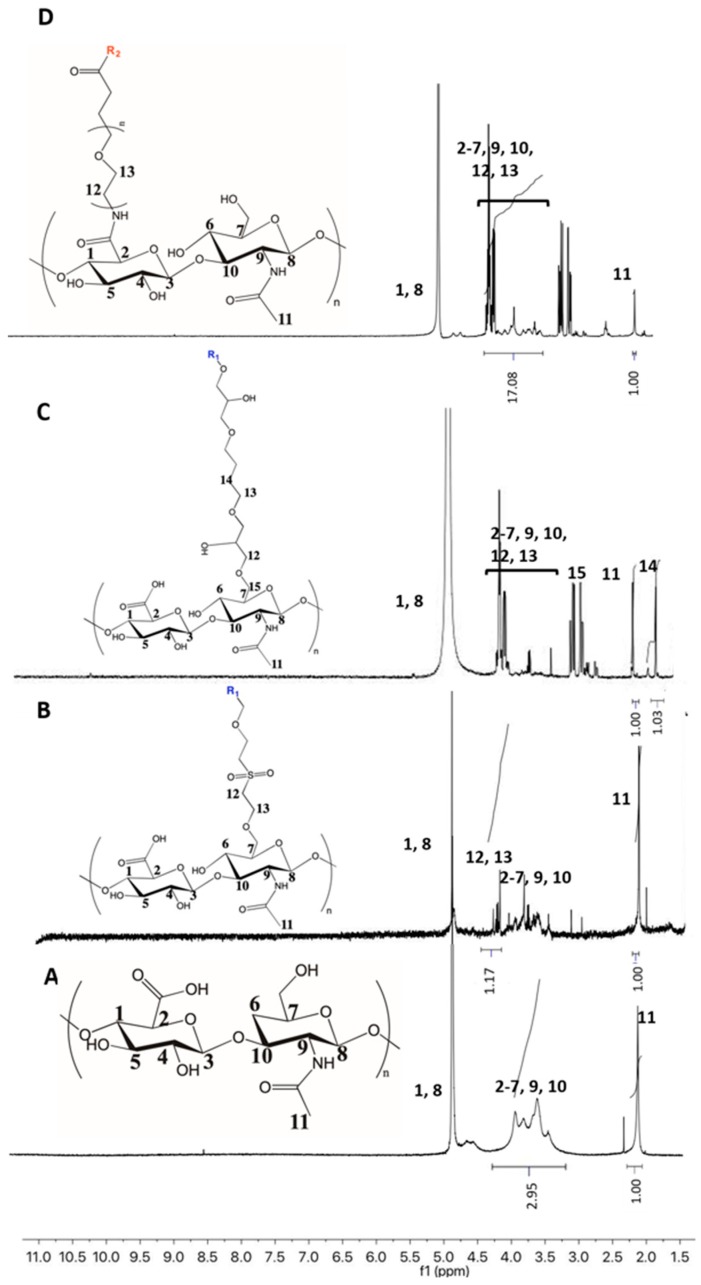
Nuclear magnetic resonance (^1^H-NMR) spectra of hyaluronic acid (2.1 MDA) in D_2_O (**A**) and HA nanogels prepared by its crosslinking in microemulsion with (**B**) DVS, (**C**) PEGBNH_2_, and (**D**) BDDE in a molar ratio HA: crosslinker of 1:1.

The ^1^H-NMR spectra of obtained nanogels dispersions revealed the incorporation of the crosslinking agents and made possible the estimation of the degree of modification of HA and, subsequently, the degree of crosslinking of the nanogels. A typical spectrum of pristine HA is shown in Figure 3A. A broad multiplet around 3.2 and 4.2 ppm corresponds with the signals of the protons in the sugar rings, while the resonance signal at 4.5 ppm is assigned to the two anomeric protons, H1 and H8. The clearly defined signal at 1.9 ppm corresponds to the methyl protons of the *N*-acetyl groups of HA. This signal was used as the reference to calculate the modification degree after crosslinking during nanogel preparation [29]. The colloidal nature of nanogels restricts the intensity of the resonance of the signals in the NMR spectra with respect to pure HA, as can be observed in Figure 3.

Regarding DVS crosslinking, the appearance of new signals around 4 ppm, corresponding to the introduced –CH_2_-moieties of the DVS molecule could be observed. Since these alkyl protons are adjacent to oxygen and sulphur atoms, their resonance leads to a multiplet at higher chemical shifts than alkyl protons of initial HA, enabling their integration. The degree of modification was determined by the comparison of the integration of DVS alkyl protons (H12–13, 4.0 ppm) with that of the acetamide moiety of the *N*-acetyl-*D*-glucosamine residue of HA (1.9 ppm), which indicated a 48% incorporation of DVS to the HA sugar unit.

In the case of BDDE crosslinking, a clearly differentiated signal was observed at 1.5 ppm that is ascribed to the alkyl protons of the BDDE molecule (H14) adjacent to alkyl protons. In addition, the resonance of alkyl protons of BDDE next to oxygen atoms (H12 and H13) at 3.6–4.2 ppm could also be observed for the crosslinked nanoparticles. Nevertheless, the substitution degree in this case was easily determined by calculating the relative integration of alkyl protons appearing at 1.5 ppm of BDDE and the methyl protons of the *N*-acetyl group of HA (1.9 ppm), and a value of 48% was obtained. The BDDE linkage to the HA backbone could be confirmed by the resonance signals of H15 protons at 2.9–3.0 ppm that appeared shifted from those smaller signals of the unreacted BDDE (2.6–2.8 ppm). These last peaks were not significant enough for quantitative determinations.

The PEG grafted onto the activated carboxylic acid of HA dealt with the appearance of a new broad resonance signal around 3.3–4.0 ppm being superimposed with the peaks of the native HA, corresponding to the –CH_2_ groups of the PEG backbone. The substitution degree could be determined by comparing the ratios of peak integrals from these alkyl moieties with respect to the methyl protons of the *N*-acetyl group of HA (1.9 ppm) prior and after the modification reaction with PEGBNH_2_. The modification degree, so determined, corresponded to a 1.2%. Thus, HA modification reactions by means of hydroxyl groups, such as those carried out with DVS and BDDE, led to similar modification degrees that were higher than those that implied reaction by the carboxylic acid groups of HA. It seems that the limitation in the number and accessibility of the carboxylic group of HA restricts the efficiency of the amidation reaction for crosslinking [43].

**Figure 4:**
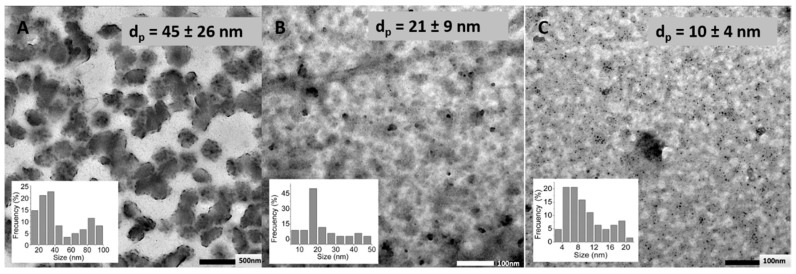
Transmission electron microscopy (TEM) microphotographs of (**A**) DVS, (**B**) BDDE, and (**C**) PEGBNH_2_, crosslinked (1:1, HA:crosslinker molar ratio) HA (2.1 MDA) nanoparticles, average particle sizes (d_p_), and particle size distributions.

TEM images (Figure 4) revealed that prepared nanogels show spherical morphology for all the crosslinking agents employed and show no significant agglomeration. Crosslinking with DVS led, in the dried state, to higher particle size (45 nm) than crosslinking with BDDE (21 nm) or PEGBNH_2_ (~10 nm). This fact could be related to the hydrophobic nature of the DVS crosslinking agent that may lead, as a consequence of hydrophobic–hydrophobic interactions, on the one hand, to more compact nanogels and, on the other hand, to the aggregates of the particles. On the contrary, the lower particle sizes in the dried state, observed in TEM photographs for nanogels crosslinked with PEG, could be ascribed to the low crosslinking degree measured for these nanoparticles.

The hydrodynamic diameters of swollen nanogels were determined by DLS measurements as the function of the external pH. For all the studied networks, nanogels showed a pH-responsive volume transition according to the pKa value of pristine HA. It is noteworthy that the pH-sensitive swelling of HA nanogels has been reported for multifunctionalized HA derivatives, such as the use of the pH labile crosslinker [44]. However, to the best of our knowledge, investigations on the direct pH response of chemically crosslinked HA nanogels have not been addressed.

**Figure 5:**
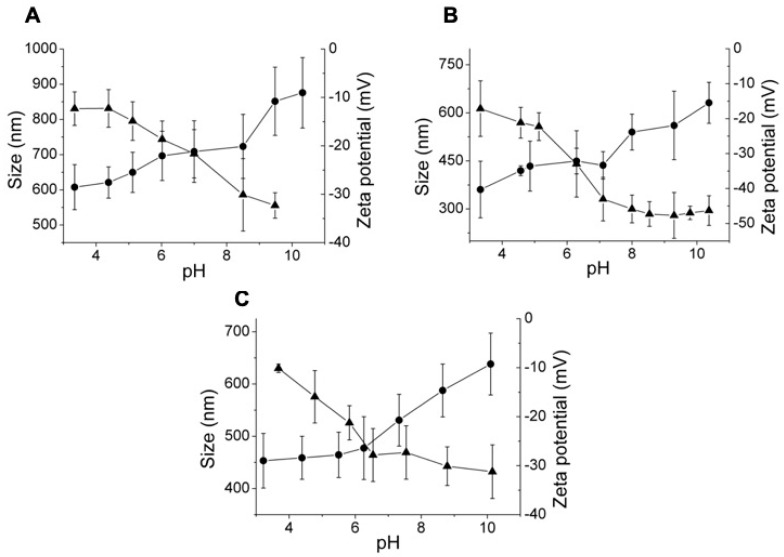
Variation of the hydrodynamic diameter (●) and the zeta potential (▲) of HA (2.1MDA) nanogels prepared by crosslinking (1:1, HA:crosslinker molar ratio) with (**A**) DVS, (**B**) BDDE, and (**C**) PEGBNH_2_.

As it is shown in Figure 5, the average particle diameter increases for all the studied systems when the external pH increases above the apparent pKa value of the nanoparticles. The carboxylic groups deionization that takes place since pH value is increased (pH > 5) promotes repulsive electrostatic interactions between –COO^−^ moieties, resulting in higher hydrodynamic diameters. This progressive deprotonation of carboxylic acids groups at high pH values was corroborated by electrokinetic potential (ξ potential) measurements of the dispersions of the nanogels, varying the pH of the medium. Indeed, the ξ-potential decreased for all the cases in the range of pH from 4 to 8.

Zeta potential measurements also corroborated the modification of carboxylic moieties by a reaction with PEGBNH_2_. The amidation reaction slightly reduced the number of carboxylate groups of HA nanogels and, as a consequence, the number of negative surface charges were diminished; ξ = −15 mV was measured at pH = 4 for HA nanogels modified by hydroxylic groups (Figure 5), while only ξ = −8 mV was displayed for PEGBNH_2_ crosslinked HA nanogels at the same pH value.

In addition, a clear effect of the chemical structure of the crosslinking agent on the particle size of the obtained nanogels in the swollen state could also be observed. The swelling degree of the network, as a consequence of the pH variation of DVS crosslinked nanogels (44%), was similar to the particle size of the nanogels crosslinked with PEGB(NH_2_) (35%), and both were lower than that of the BDDE crosslinked particles (75%). The hydrophobic and rigid structure of DVS led to larger particles, regardless of the pH of the medium, compared to the other crosslinkers, and it seems to limit the swelling of HA nanogels leading to low swelling capacity. In contrast, nanogels modified with PEGBNH_2_ showed a low swelling capacity, despite the long chain length and hydrophilic and flexible nature of this crosslinking agent. In this case, the low crosslinking grade promotes a low-swelling capacity, derived from the limited crosslinking efficiency. In addition, chemical modification of the carboxyl groups leads to a slightly reduced negative charge repulsion of HA chains, which may have a significant decreasing effect on nanogel pH-responsive swelling.

The swelling capacity of studied systems was also calculated by comparing the particle diameters measured in the dried state (TEM) and in the swollen state (pH = 4.0) of the nanogels, to evaluate the effect of the hydrophilicity of the crosslinking agents, regardless of the ionic effect. Swelling factors of 12%, 17%, and 47% could be determined for DVS, BDDE, and PEGBNH_2_, respectively, as expected, according to the differences on their chemical structure above described, and unlike the measured pH-responsive swelling degrees. Certainly, the swelling induced in an ionic network is the accumulative effect of the mixing, elastic, and ionic contributions. In the case of PEGBNH_2_, crosslinking reduces ionic contribution due to the decrease of ionisable groups in the network, but at the same time provokes the enhancement of the elastic force derived from its long chain length and hydrophilic and flexible nature that, in this case, governs the swelling of the network from the dried state and leads to high swelling grades, despite the low dimensions in the absence of water.

Therefore, it could be concluded that the election of the crosslinking agent plays a crucial role in the swelling capacity of the obtained nanogels and that the nanogels crosslinked with BDDE resulted in being the most interesting pH-responsive networks that showed the largest swelling by external pH change.

### 3.3. Effect of HA Molecular Weight and BDDE Content

HA, with two different molecular weights of 2.1 MDa (HMW) and 751 kDa (LMW), and three BDDE contents (0.2, 1, and 10 equivalents), were employed in the preparation of the nanogels crosslinked with BDDE in order to analyse the effect of these parameters on the properties of obtained nanoparticles. High crosslinking yields (>90%) could be determined by ^1^H-NMR in all the samples, except in the cases in which the 1:10 HA:BDDE feed ratio was used, because the excess of the crosslinking agent led to a misleading integration of NMR signals, as a consequence of the considerable amount of unreacted BDDE. This limits the applicability of BDDE highly-concentrated feeds in the preparation of HA nanogels.

**Figure 6:**
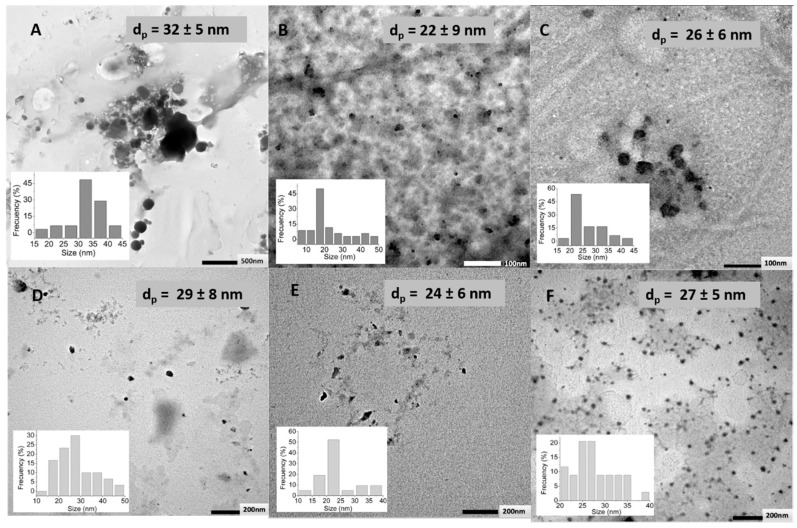
TEM microphotograph of (**A**) HA (LMW)–BDDE 1:0.2, (**B**) HA (LMW)–BDDE 1:1, (**C**) HA (LMW)–BDDE 1:10, (**D**) HA (HMW)–BDDE 1:0.2, (**E**) HA (HMW)–BDDE 1:1, and (**F**) HA (HMW)–BDDE 1:10 nanoparticles average particle sizes (d_p_) and particle size distributions.

TEM microphotographs did not show significant differences on the particle size of dried nanogels nor when the molecular weight of HA was varied, neither was the BDDE content changed, as can be observed in Figure 6.

**Figure 7:**
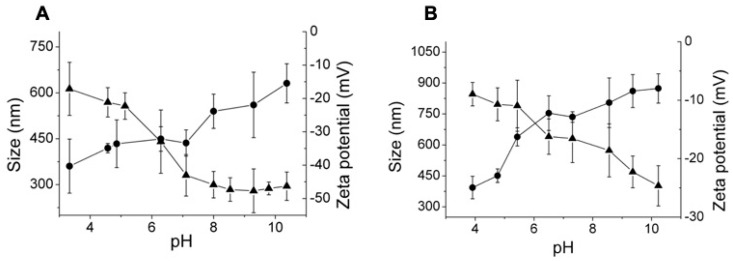
Hydrodynamic diameters (●) and zeta potential (▲) as a function of external pH for HA nanogels obtained by crosslinking with BDDE with (**A**) high molecular weight and (**B**) low molecular weight HA.

Moreover, as it is shown in Figure 7, HA nanogels with high and low molecular weight led to similar swelling grades from the dried state to the swollen state at pH = 4.0 (17.4 and 18.0% for high and low molecular weight HA, respectively). However, slightly greater swelling could be observed in the swelling degree as a consequence of the pH variation (75% and 94% for high and low molecular weight HA, respectively). This data could be explained by the physical entanglements characteristics of high molecular weight macromolecular chains that restrict the swelling of the nanogels [45,46]. Certainly, these differences are also related to the crosslinking reaction because the crosslinking grades determined by ^1^H-NMR spectroscopy were slightly higher (78.0%) for high molecular weight HA than for low molecular weight HA (73%) (Supplementary Figure S1A,B).

**Figure 8:**
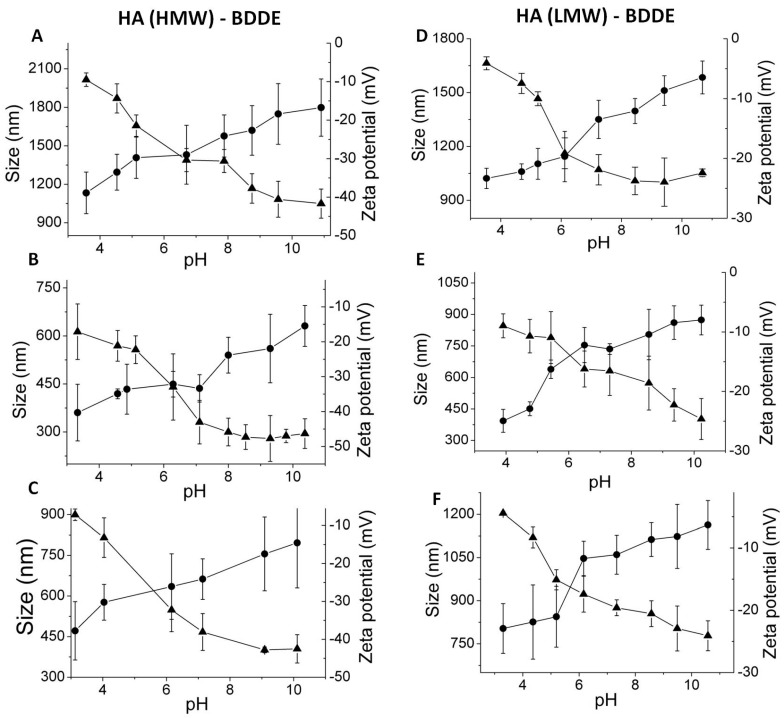
Hydrodynamic diameters (●) and zeta potential (▲) as a function of external pH for HA nanogels obtained by the crosslinking of HA (high molecular weight) with BDDE at different ratios: (**A**) HA (HMW)–BDDE 1:0.2, (**B**) HA (HMW)–BDDE 1:1, and (**C**) HA (HMW)–BDDE 1:10 and obtained by crosslinking of HA (low molecular weight) with (**D**) HA (LMW)– =BDDE 1:0.2, (**E**) HA (LMW)–BDDE, and (**F**) HA (LMW)–BDDE 1:10.

In addition, a clear variation in the zeta potential of the nanogels prepared with different molecular weights of HA could be observed. As Figure 7 and Figure 8 show, nanogels prepared with high molecular weight HA also displayed more negative charge than nanogels prepared with low molecular weight HA. This fact is in accordance with the more negative charge of the uncrosslinked high molecular weight HA molecules (−18 mV at pH = 4 and −65 mV at pH = 8) in comparison with low molecular weight HA (−14 mV at pH = 4 and −45 mV at pH = 8).

Increasing BDDE content was employed for the preparation of HA (low and high molecular weight) nanogels. Obtained DLS measurements showed, regardless of the molecular weight of HA, a significant effect when a low BDDE content was added on the hydrodynamic diameters of the nanogels. Networks prepared with 0.2 equivalents of BDDE showed particle sizes on the micrometric scale and hydrodynamic diameters 2.2 times higher on the range of pH than nanogels with 1:1 and 1:10 HA:BDDE feed ratio (Figure 8). Thus, it seems that the effect of a low reticulation degree (1:0.2 HA:BDDE) of the network favoured swelling properties (swelling by pH change and swelling from the dried to the swollen state) of the nanogels (Figure 7, Table 1). As expected, high crosslinking concentrations (1:1 HA:BDDE) lead to a higher elastic contribution to the swelling of the network that restricts its particle size. However, as it has also been demonstrated in the literature that, for certain crosslinking values, this effect is cancelled and countered by the physical steric hindrance added by crosslinker molecules [47]. It seems that this was the case of the nanogels prepared with high concentrations of crosslinking, 1:10, HA:BDDE initial ratio.

## 4. Conclusions

Hyaluronic acid was successfully crosslinked with the biocompatible crosslinkers divinyl sulfone (DVS), 1,4-butanediol diglycidyl ether (BDDE), and poly(ethylene glycol) bis(amine) (PEGBNH_2_) by W/O microemulsion leading to ultrafine nanogels (<50 nm TEM). The prepared nanogels displayed a pH-responsive swelling according to the pKa of the non-crosslinked hyaluronic acid, this is, the hydrodynamic diameter of the nanogels colloid dispersions increased by increasing the pH from 4 to 8. The QELS analysis showed how the swollen dimensions can be ranged from the micro to the nanoscale by varying the crosslinking degree for BDDE crosslinked gels. Despite the molecular weight not showing a significant effect on the particle size of dried nanogels, the physical entanglements derived from a high chain length led to restricted swollen particle sizes. In addition, the swelling capacity from the dried to the unionized swollen state, and that derived by changes on the external pH, clearly depended on the chemical structure of the crosslinking agent. The nanogels crosslinked with BDDE were the most interesting pH-responsive networks conducting the largest swelling as a response to external pH change. The pH-responsive swelling of the studied nanogels make them attractive candidates as degradable and biocompatible nanocarriers for a large variety of biomedical applications.

## Figures and Tables

**Table 1:** Swelling degree (%) and crosslinking percentage of HA nanogels prepared with different initial molecular weights.

HA Molecular Weight	BDDE Equivalents	Swelling Degree (%) (Dried-swollen pH = 4)	Swelling Degree (%) (∆pH)	Crosslinking (%) ^1^
2.1 MDa	1–0.2	28	59	15
	1–1	17	75	78
	1–10	21	38	-
751 KDa	1–0.2	35	53	22
	1–1	18	94	73
	1–10	29	36	-

^1^ Determined by ^1^H-NMR.

## Supplementary Materials

The following are available online at https://www.mdpi.com/2073-4360/11/4/742/s1, Figure S1: ^1^H-NMR spectra of (A) HA (HMW)–BDDE 1:1 and (B) HA (LMW)–BDDE 1:1, Figure S2: ^1^H-NMR spectra of HA nanogels prepared by crosslinking in microemulsion with HA (HMW) and BDDE in molar relation of (A) 1:0.2, (B) 1:1, and (C) 1:10, Figure S3: ^1^H-NMR spectra of HA nanogels prepared by crosslinking in microemulsion with HA (LMW) and BDDE in molar relation of (A) 1:0.2, (B) 1:1, and (C) 1:10.
