# Supplementary material for: Synthesis and Characterization of Covalently Crosslinked pH-Responsive Hyaluronic Acid Nanogels: Effect of Synthesis Parameters
Source: Polymers (Basel). 2019 Apr 24;11(4):742. doi: 10.3390/polym11040742 (PMC6523595; doi:10.3390/polym11040742)
Supplement: Supplementary file 1 [file polymers-11-00742-s001.pdf]

polymers

MDPI

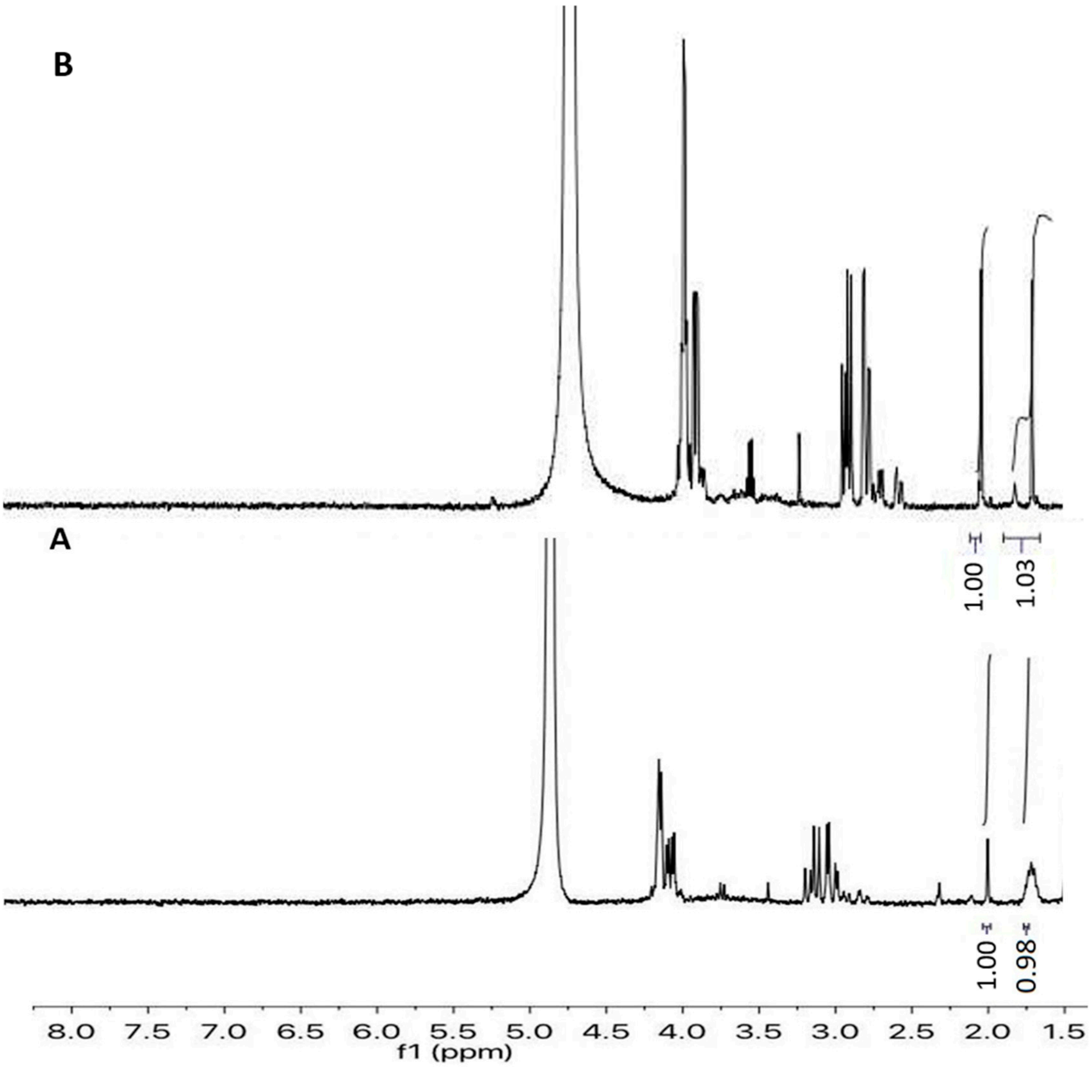

Figure S1. ^{1}H-NMR spectra of (A) HA (HMW)-BDDE 1:1 and (B) HA (LMW) – BDDE 1:1

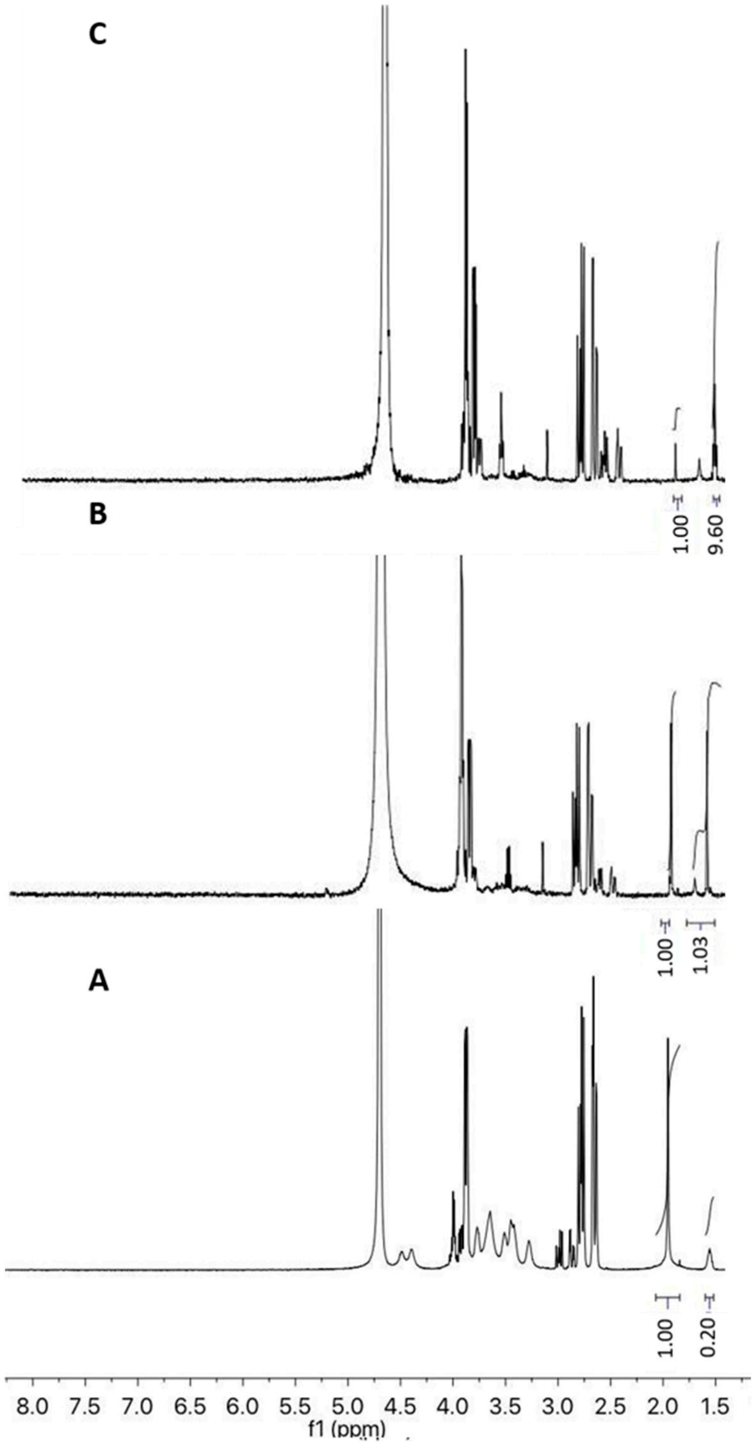

Figure S2. ^{1}H NMR spectra of HA nanogels prepared by its crosslinking in microemulsion with HA (HMW) and BDDE in molar relation of (A) 1:0.2, (B) 1:1 and (C) 1:10

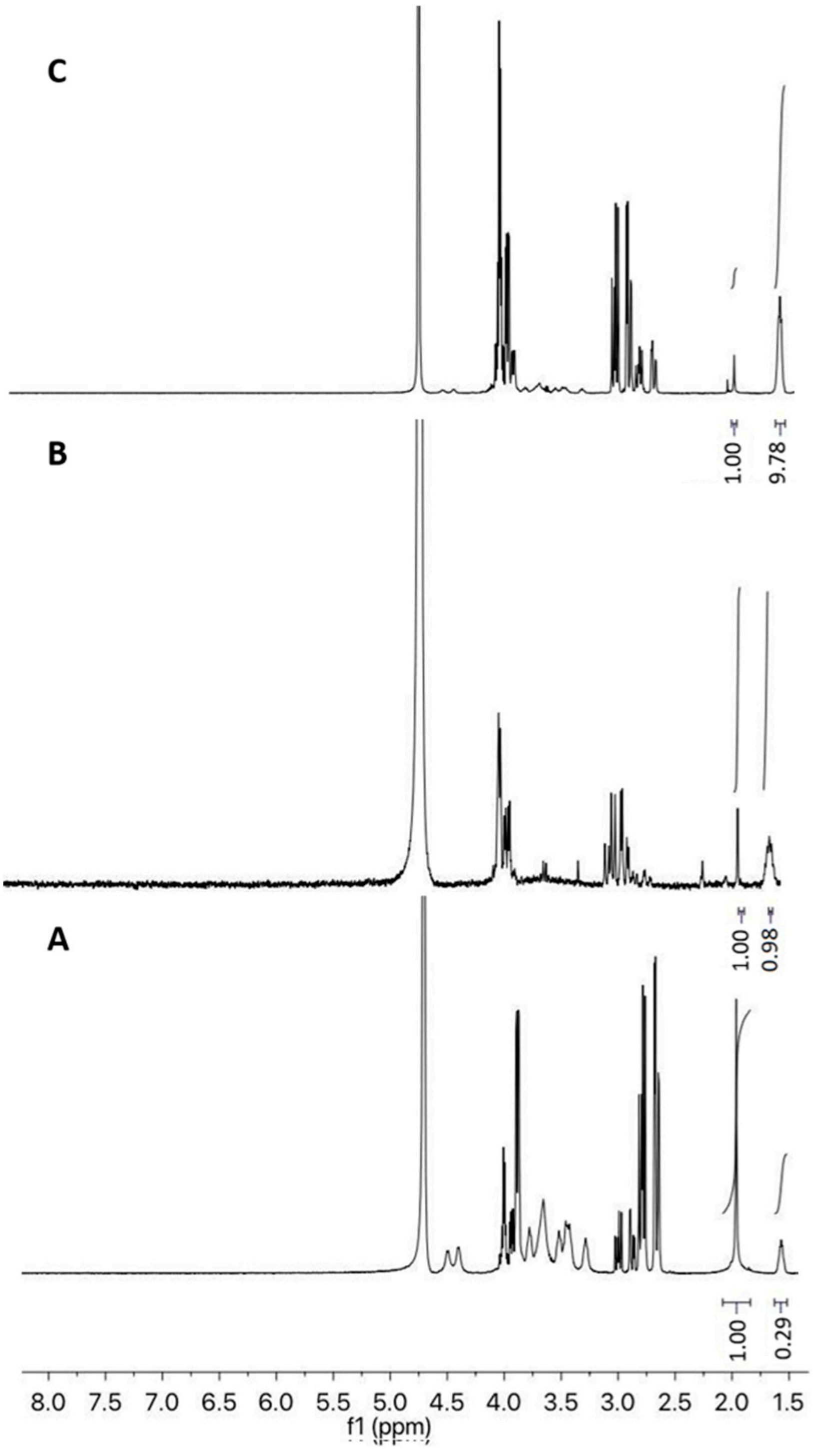

Figure S3. ^{1}H NMR spectra of HA nanogels prepared by its crosslinking in microemulsion with HA (LMW) and BDDE in molar relation of (A) 1:0.2, (B) 1:1 and (C) 1:10
